# Der Stellenwert der Psychiatrie in der Versorgung und Forschung von Post-COVID

**DOI:** 10.1007/s00115-026-01951-w

**Published:** 2026-03-17

**Authors:** Martin Walter, Kristina Adorjan, Malek Bajbouj, Jürgen Deckert, Sabine Köhler, Klaus Lieb, Josef Priller, Claudia Schillig, Anna-Karina Schomburg, Georg Schomerus, Meryam Schouler-Ocak, Andreas Meyer-Lindenberg

**Affiliations:** 1 Universitätsklinik für Psychiatrie und Psychotherapie, Universitätsklinikum Jena, Philosophenweg 3, 07743 Jena, Deutschland; 2 Klinik für Psychiatrie und Psychotherapie (UPD), Universität Bern, Bern, Schweiz; 3 Klinik für Psychiatrie und Psychotherapie, CCM, Charité, Universitätsmedizin Berlin, Berlin, Deutschland; 4 Institut für Klinische Epidemiologie und Biometrie, Julius-Maximilians-Universität Würzburg, Würzburg, Deutschland; 5 Zentrum für Psychische Gesundheit, Klinik und Poliklinik für Psychiatrie, Psychosomatik und Psychotherapie, Uniklinikum Würzburg, Würzburg, Deutschland; 6 Praxis für Psychiatrie und Psychotherapie, Jena, Deutschland; 7 Deutsche Gesellschaft für Psychiatrie und Psychotherapie, Psychosomatik und Nervenheilkunde e. V., Berlin, Deutschland; 8 Klinik für Psychiatrie und Psychotherapie, Universitätsmedizin Mainz, Mainz, Deutschland; 9 Klinik und Poliklinik für Psychiatrie und Psychotherapie, TUM Universitätsklinikum, München, Deutschland; 10 Zentralinstitut für Seelische Gesundheit, Klinik für Psychiatrie und Psychotherapie, Medizinische Fakultät Mannheim, Universität Heidelberg, Mannheim, Deutschland; 11 Institut für Ethik und Geschichte der Medizin, Universitätsmedizin Göttingen, Göttingen, Deutschland; 12 Klinik und Poliklinik für Psychiatrie und Psychotherapie, Universitätsklinikum Leipzig, Leipzig, Deutschland

**Keywords:** Neuropsychiatrische Symptome, Interdisziplinäre Versorgung, Post-COVID-19, Therapieoptionen, Klinische Forschung

## Abstract

Postinfektiöse Syndrome wie Post-COVID sind durch die COVID-19-Pandemie in den Fokus gerückt. Das weite Spektrum von Beschwerden umfasst neben Kopf- und Gliederschmerzen, Palpitationen, Hautveränderungen und Kurzatmigkeit auch neuropsychiatrische Symptome wie Fatigue, Gedächtnis- und Konzentrationsstörungen sowie Angst und affektive Symptome. Die Ätiologie und Pathophysiologie der Symptome sind noch weitgehend unbekannt. Das Erleben einer schwer behandelbaren und potenziell chronischen Erkrankung stellt eine erhebliche Belastung für Betroffene dar, vor allem auch für Menschen mit psychischen Vorerkrankungen. Es ist unbedingt erforderlich, eine sorgfältige Abgrenzung zu anderen psychischen Erkrankungen vorzunehmen. Um Betroffene bei der Bewältigung der Folgen von Post-COVID adäquat zu unterstützen, ist psychiatrische Expertise zwingend nötig. Dieser Artikel zeigt auf, welchen Beitrag die Psychiatrie bei der Diagnostik und Behandlung von Post-COVID leisten kann und wie sie zu einem besseren Verständnis des Krankheitsbildes beiträgt. Es werden Behandlungsansätze und Forschungschancen diskutiert. Fachpolitische und gesellschaftliche Forderungen nach einem ganzheitlichen Ansatz, der eine interdisziplinäre Herangehensweise erfordert und sowohl klinische und wissenschaftliche Expertise als auch die Perspektive der Betroffenen miteinbezieht, werden erläutert.

Postinfektiöse Syndrome wie Post-COVID stellen eine große Herausforderung für die Forschung und Behandlung dar. Patientinnen und Patienten sind von zumeist chronischen und schwerwiegenden Symptomen wie Myalgien, Kopf- und Gliederschmerzen, aber auch von Fatigue, Angst, Konzentrations- und Gedächtnisstörungen betroffen. Die Ursachen der Symptome sind noch nicht geklärt. Dieser Beitrag soll verdeutlichen, wie die Psychiatrie und Psychotherapie helfen können, postinfektiöse Syndrome nicht nur besser zu verstehen, sondern auch diagnostische Verfahren und Behandlungsoptionen zu erforschen und weiterzuentwickeln.

## Der Stellenwert neuropsychiatrischer Symptome von Post-COVID

Die COVID-19-Pandemie hat postinfektiöse Syndrome wie Post-COVID in den Fokus der Aufmerksamkeit gerückt. Konservativen Schätzungen zufolge entwickeln etwa 2–10 % der Betroffenen nach einer SARS-CoV-2-Infektion länger andauernde Symptome und sind über viele Monate teilweise erheblich eingeschränkt [1, 2]. Ein kleinerer Teil der Betroffenen entwickelt ein schweres, chronisches, postinfektiöses Syndrom. Die Ausprägung des Beschwerdebildes reicht von einer leichten und vorübergehenden Minderung der Lebensqualität bis hin zu langdauernden, schweren Einschränkungen, die dem klinischen Bild der myalgischen Enzephalomyelitis/chronisches Fatigue-Syndrom (ME/CFS) entsprechen.

Post-COVID umfasst ein weites Spektrum an Beschwerden. Häufig kommt es zu Kopf- und Gliederschmerzen, Palpitationen, Kurzatmigkeit, Hautveränderungen oder Haarausfall [3]. Auch neuropsychiatrische Symptome wie Fatigue, kognitive Einschränkungen, Konzentrationsstörungen [1], Angst und affektive Symptome [4] sind verbreitet. Ähnlich wie bei ME/CFS kommen spezifische Symptome wie Belastungsintoleranz („exercise intolerance“), „post-exertional malaise“ (PEM) sowie Ein- und Durchschlafstörungen, Müdigkeit und Erschöpfbarkeit vor [2].

Menschen mit psychischen Vorerkrankungen haben ein erhöhtes Risiko, an Post-COVID zu erkranken [3]. Das Erleben einer schwer behandelbaren, potenziell chronischen und stark beeinträchtigenden Erkrankung stellt aber auch unabhängig von psychischen Vorerkrankungen eine erhebliche psychische Belastung dar. Bestimmte Symptomkomplexe, z. B. Erschöpfbarkeit und kognitive Einschränkungen, stellen eine differenzialdiagnostische Herausforderung dar, insbesondere, wenn sie zunächst mithilfe von Screeninginstrumenten erhoben werden. Die wenigen Studien, die Daten über Migrantinnen und Migranten und ethnische Minderheiten enthalten, deuten ferner darauf hin, dass diese Gruppe wenig untersucht und unverhältnismäßig stark von Post-COVID betroffen sein könnte [4].

Menschen mit Post-COVID erfahren häufig Stigmatisierung. Über 80 % der Betroffenen berichten, wegen ihrer Erkrankung im persönlichen Umfeld, aber auch im Behandlungskontext abgewertet worden zu sein [5–9]. Zentral für das Stigmaerleben ist die Zuschreibung der Symptome auf psychische (Mit‑)Ursachen, die als abwertend und trivialisierend erlebt wird [10]. Dies ist besonders folgenschwer, wenn dadurch Therapiemaßnahmen wie z. B. leitliniengerechte Pacing-fokussierte Psychoedukation unterbleiben oder falsche Therapieentscheidungen getroffen werden. Eine sorgfältige Abgrenzung von anderen psychischen Erkrankungen ist Voraussetzung dafür, die jeweils korrekte Behandlung anzubieten. Dies unterstreicht die Bedeutung interdisziplinärer Forschung und Behandlung, die psychiatrische Expertise zeitnah mit einbezieht.

## Psychiatrische Perspektive auf Krankheitsgenese

Ätiologie und Pathophysiologie der bekannten Symptome sind noch weitgehend unbekannt, auch wenn der Wissensstand hierzu ständig wächst [10]. Nach wie vor wird über Krankheitsmechanismen aus der Perspektive einzelner Disziplinen kontrovers diskutiert. Dabei wird nicht selten im Sinne einer Dichotomie argumentiert, in der „somatische“ streng von psychobiologischen Erklärungsmodellen getrennt werden. Unter dem Gesichtspunkt einer optimalen, individualisierten Therapie birgt diese Dichotomie Gefahren, weil reziproke Wechselwirkungen unterschätzt werden und möglicherweise vorliegende psychiatrisch behandelbare Störungen unbehandelt bleiben. Umgekehrt kann eine vorschnelle Einordung der Symptomatik als „psychogen“ ebenfalls zu falschen Behandlungsentscheidungen führen. Als psychiatrische Fachgesellschaft vertreten wir deshalb ein ganzheitliches Krankheitsverständnis, das unterschiedliche Ätiologien in unterschiedlichen Gewichtungen zulässt [11].

Dieses ganzheitliche Verständnis basiert auf einer Vielzahl bidirektionaler Evidenzen bei anderen Krankheitsbildern, in denen sowohl das Auftreten psychiatrischer Syndrome bei (auto-)immunologischen Erkrankungen als auch immunologische Veränderungen bei „klassischen“ psychiatrischen Erkrankungen gezeigt werden konnten. Paradigmatische Beispiele sind Autoimmunenzephalitiden, bei denen Veränderungen von Verhalten und Befinden durch Antikörper verursacht sind. Ein weiteres, insbesondere für das Verständnis von Post-COVID relevantes Syndrom ist das „sickness behaviour“ [12, 13], bei dem sowohl im Tiermodell als auch beim Menschen durch eine systemische Entzündungsreaktion Symptome wie Appetitlosigkeit, Depressivität, Ängstlichkeit sowie eine erhöhte Schmerzempfindlichkeit beobachtet werden können. Solche Symptome werden im Wesentlichen durch systemische und zentralnervöse Wirkungen inflammatorischer Zytokine verursacht, die schon in geringen Konzentrationen Verhalten und Befinden beeinflussen [14].

Umgekehrt gibt es bei Personen mit affektiven Störungen Hinweise auf ein Wechselspiel zwischen inflammatorischen Prozessen und depressiven Kernsymptomen wie niedergedrückte Stimmung, Schlafstörungen und Appetitstörungen („inflammatorische Depression“; [15]). Wie die Symptome im Rahmen von Post-COVID entstehen und ob Subtypen existieren, die vergleichbar zu Autoimmunenzephalitiden (Autoimmunprozesse führen zu psychischen Symptomen) zur sog. immunometabolischen Depression (psychische Symptome führen zu inflammatorischen Prozessen) oder zu zentralnervösen Auswirkungen systemischer Inflammation bei gestörter Blut-Hirn-Schranke [16] eingeordnet werden, ist bisher unklar. Obwohl es zahlreiche Befunde zu biologischen Markern gibt, die deutliche Gruppenunterschiede zwischen gesunden Probanden und Menschen mit Post-COVID aufweisen, gibt es noch keine diagnostischen Marker auf individueller Ebene. Diese sind für die dringend erforderliche Entwicklung neuroimmunologischer Therapien der Postinfektionssyndrome von großer Bedeutung.

Es gibt Hinweise, dass neben neuroimmunologischen Prozessen auch psychosoziale Faktoren eine Rolle bei Post-COVID spielen [17]. Neben biologischer (z. B. genetischer) Vulnerabilität spielen vermutlich auch sozioökonomische Faktoren wie Bildung, Einkommen und Beschäftigungsstatus sowie die Möglichkeit einer Belastungsreduktion im Alltag zur Vermeidung von PEM eine Rolle, auch wenn ihre Interaktion mit den spezifischen Krankheitsprozessen bei Post-COVID noch unklar ist. Das heterogene klinische Krankheitsbild ist deshalb möglicherweise auch durch ein komplexes Zusammenspiel interdependenter biologischer, psychologischer und sozialer Faktoren geprägt [18].

## Behandlungsansätze

Behandlungsansätze müssen psychische Erkrankungen, welche nach einer COVID-Infektion auftreten oder wieder auftreten, von Syndromen abgrenzen, welche mit oder im Rahmen von Post-COVID auftreten (z. B. depressive Symptome), jedoch zum Zeitpunkt der Behandlungsnotwendigkeit die diagnostischen Kriterien für eine dadurch definierte psychische Erkrankung nicht erfüllen. Für erstere (z. B. Depressionen und Angsterkrankungen) gelten unabhängig vom Auftreten einer SARS-COV-2-Infektion die AWMF-Leitlinien zur Behandlung dieser psychischen Erkrankungen. Nur diesen Betroffenen können leitliniengerecht innerhalb der jeweiligen Zulassung psychopharmakologische Therapien angeboten werden [19].

Nach dem Therapiekompass des Bundesinstituts für Arzneimittel und Medizinprodukte (BfarM) können zusätzlich indikationskonsistente symptomatische Ansätze, z. B. bei Schlafstörungen, genutzt werden, welche in etwa 50 % der Fälle und auch unabhängig von einer affektiven Störung auftreten [20, 21]: Für diese stehen bereits jetzt, eine differenzialdiagnostische Einordnung vorausgesetzt, spezifische Behandlungsmethoden zur Verfügung. Die Wirksamkeit einzelner Verfahren, z. B. kognitive Verhaltenstherapie [22] oder pharmakologische Ansätze für ängstliche oder depressive Syndrome im Rahmen von Post-COVID, muss gegenwärtig noch untersucht werden.

Es gibt wenige Daten und entsprechend wenige Empfehlungen für die deutlich größere Gruppe mit zeitlich fluktuierenden depressiven Symptomen, die nicht die Kriterien einer depressiven Episode erfüllt. Für die Behandlung neuropsychiatrischer Symptome bei Post-COVID sind bislang nur einzelne, in Deutschland derzeit nicht erstattungsfähige Substanzen wie Vortioxetin [23] auf hohem Evidenzlevel untersucht worden, die Effekte auf das Funktionsniveau und die kognitive Symptomatik gezeigt haben. Empfehlungen für den Einsatz von Agomelatin [24] vor allem bei Fatigue beruhen in Teilen auf Erfahrungen bei ME/CFS außerhalb von Post-COVID. Im Falle einer Berücksichtigung der Empfehlungen der BfarM-Expertenkommission und entsprechender Marktverfügbarkeit könnte jedoch mittelfristig eine erstattungsfähige pharmakologische Off-Label-Behandlung bei depressiven und kognitiven Einschränkungen bei Post-COVID ohne diagnostizierte Depression verfügbar werden [25–27].

Darüber hinaus existieren Verfahren, deren klinischer Einsatz aufgrund unzureichender Evidenz noch nicht empfohlen werden kann: So werden erste positive Ergebnisse von hyperbarer Sauerstofftherapie (HBO) bei neuropsychiatrischem Post-COVID berichtet [28, 29]. Weitere Substanzen werden außerhalb ihrer eigentlichen Indikation und Dosierung derzeit noch untersucht, u. a. Low-dose-Naltrexon (LDN; [30]). Dies gilt auch für noninvasive Hirnstimulationstechniken wie die transkutane Vagusnervstimulation (tVNS), deren Rationale es ist, der bei Post-COVID beschriebenen Dysautonomie entgegenzuwirken [31].

Ähnlich wie bei Medikamenten gibt es noch keine zugelassenen Psychotherapieverfahren für Post-COVID. Ein randomisierter Trial liefert Hinweise, dass kognitive Verhaltenstherapie Fatigue im Rahmen von Post-COVID günstig beeinflussen kann, die meisten Studien sind allerdings unkontrolliert [22]. In Abwesenheit effektiver kausaler Therapiestrategien sind damit Krankheitsbewältigung und, beim Vorliegen einer PEM, das Erlernen von Pacing wichtige Therapieziele.

Gegenüber affektiven Symptomen wurden neuropsychologisch objektivierbare kognitive Defizite im longitudinalen Verlauf als stabil, wenn auch bei vielen als rückläufig beschrieben [25–27]. Für Maßnahmen zur Förderung der kognitiven Erholung bei Post-COVID fehlt aktuell noch höherwertige Evidenz, wobei kognitives Training zeitlich uneingeschränkt verordnet werden kann. In Anbetracht des sehr frühen Stands der Forschung fehlen spezifische Untersuchungen zu sprach- und kulturgebundenen Verständigungsproblemen. Hier besteht erheblicher Forschungsbedarf, um diversitäts- und diskriminierungsfreie Zugangsmöglichkeiten für Betroffene zu schaffen.

## Offene Fragen und Forschungschancen

Bezüglich der Pathophysiologie von Post-COVID verdichten sich Befunde, dass niedriggradiges Entzündungsgeschehen [7, 8], inflammatorisch getriggerte mikrovaskuläre Pathologien und Dysautonomie [16, 32] eine Rolle spielen. Dennoch ist die Erkrankung in ihrer Komplexität noch nicht entschlüsselt. Ein drängendes Ziel ist, aus dem Verständnis von Pathomechanismen kausale und personalisierte Therapiestrategien abzuleiten und diese zu evaluieren. Es ist denkbar, dass in der Frühphase eines postinfektiösen Syndroms andere Therapiestrategien wirksam sind als in dessen chronifizierter Form. Insofern stellt das pandemiebedingt gleichzeitige Auftreten von Post-COVID in großer Zahl auch eine Chance dar, die Pathomechanismen der Frühphase postinfektiöser Syndrome zu untersuchen.

Weitere offene Fragen beziehen sich auf den natürlichen Verlauf und die Langzeitprognose von Post-COVID und ME/CFS und auf Faktoren, welche unterschiedliche Trajektorien innerhalb von Post-COVID vorhersagen. Forschung zu Post-COVID sollte der Heterogenität hinsichtlich der Schwere des Krankheitsbildes Rechnung tragen. Zudem liegt bei Post-COVID im Gegensatz zu präpandemischem ME/CFS eine bezüglich des auslösenden Agens und der Krankheitsdauer homogenere Patientengruppe vor, was für die Erforschung von Pathomechanismen von Vorteil ist. Eine besondere Bedeutung kommt dem Symptom der PEM bei, da es ein Alleinstellungsmerkmal von ME/CFS darstellt und die Erkrankung von symptomatisch überlappenden Syndromen abgrenzt.

Insgesamt bietet ein besseres Verständnis der Pathogenese von Post-COVID die Chance eines Transfers der Erkenntnisse auf postinfektiöse Syndrome nach anderen Erregern und hat Bedeutung für das Verständnis anderer Syndrome mit dem Leitsymptom Fatigue.

## Fachpolitische und gesellschaftliche Forderungen

Sowohl in der Forschung als auch in der klinischen Versorgung braucht es einen integrativen und interdisziplinären Ansatz, um die Vielzahl von Faktoren zu berücksichtigen, die Post-COVID prägen. Dabei sollten nicht nur biologische und psychische, sondern auch soziale und Umweltfaktoren in die Diagnose und Therapie einfließen. Ein wesentlicher Bestandteil dieses Ansatzes ist die Einbindung Betroffener in die Behandlung und Forschung. Beteiligungsmodelle sollten gefördert werden, in denen Betroffene aktiv und auf Augenhöhe in die Gestaltung von Studien und Behandlungsrichtlinien eingebunden werden. Ziel ist eine patientenzentrierte Versorgung, die sowohl die aktuelle medizinische Praxis als auch die Lebensrealität der Betroffenen berücksichtigt.

Universitätsmedizinische Postinfektionszentren im Rahmen des NUM-Infektionsnetzwerkes sollten daher einen interdisziplinären Ansatz verfolgen. Um die Versorgung von Menschen mit Post-COVID zu optimieren, ist eine enge Zusammenarbeit zwischen universitären Postinfektionszentren, ambulanten Fachärztinnen und -ärzten sowie Hausärztinnen und -ärzten unerlässlich. Durch ein vernetztes Zusammenarbeiten von vertragsärztlich tätigen Haus- und Fachärzten in Zusammenarbeit mit den Spezialambulanzen kann die Versorgungsqualität gesteigert werden. Insbesondere ist die Koordination der Versorgung und der Austausch zwischen verschiedenen Akteuren erforderlich, um die optimale fortlaufende Betreuung der Betroffenen bei derzeit häufig fehlenden kurativen Ansätzen sicherzustellen.

Die differenzielle Diagnostik psychischer Beschwerden bedarf der fachärztlich-psychiatrischen Expertise im Kontext somatischer Erkrankungen, weshalb ein gestuftes Diagnose- und Versorgungssystem unverzichtbar ist. In diesem müssen die psychischen Beschwerden beachtet, die Feststellung und Abgrenzung psychiatrischer Diagnosen und das Erreichen spezialisierter Expertise bei Vorliegen eines Post-COVID-Syndroms in einem Versorgungsnetz ermöglicht werden. Maßnahmen zur Teilhabefähigkeit müssen im Rahmen der multiprofessionellen Versorgung von Beginn an in den Fokus genommen werden. Da oft lange Krankheitsverläufe bestehen, muss auf die Langfristigkeit aller Maßnahmen geachtet werden. Durch eine multiprofessionelle Integration ärztlicher, therapeutischer und pflegerischer Kompetenzen kann ein umfassendes Behandlungsangebot für Betroffene entstehen.

Die wissenschaftliche Forschung zu Post-COVID in verschiedene Richtungen sollte gestärkt und die öffentliche Wahrnehmung von Post-COVID in den Fokus gerückt werden, um das Verständnis für die Krankheit zu verbessern. Neu etablierte, auf Translation ausgelegte Forschungsinitiativen wie das Deutsche Zentrum für psychische Gesundheit (DZPG) mit neuroimmunologischer und translationaler Expertise sind hierfür geeignet. Genehmigungsverfahren für experimentelle und klinische Studien sollten effizienter gestaltet werden, um die zeitnahe Umsetzung von Forschungsergebnissen in die Praxis zu fördern. Plattformstudien sind eine geeignete und in der Pandemie erprobte Form, die Gewinnung von Studienevidenz in die klinische Praxis zu integrieren. Eine wichtige Maßnahme ist die Etablierung einer koordinierten Forschungsförderung, die Ressort- und Ländergrenzen übergreifend zusammenarbeitet, um den Forschungsprozess zu beschleunigen. Ein erster Schritt ist mit der Gründung der Allianz für postinfektiöser Erkrankung des BMFTR und des Bundesministeriums für Gesundheit (BMG) getan.

Viele Betroffene sehen sich nicht nur mit gesundheitlichen, sondern aufgrund von Stigmatisierung auch mit sozialen und psychischen Belastungen konfrontiert. Die Forderung nach Maßnahmen zur Bekämpfung der Stigmatisierung von Post-COVID-Betroffenen ist daher ein wichtiges Thema, das nicht nur die medizinische, sondern auch die gesellschaftliche Dimension der COVID-19-Pandemie betrifft. Reduktion von Stigma bei psychischen Erkrankungen und Vermeidung stigmatisierend erlebter vorschneller, falscher oder einseitiger Einordung der Symptome als psychische Störung sind hierbei kein Wiederspruch. Voraussetzung für eine stigmafreie Behandlung sind ausreichende Ressourcen, um der Komplexität des Krankheitsbildes gerecht zu werden. Maßnahmen wie Schulungen medizinischer Fachkräfte, um Post-COVID als ernsthafte Erkrankung anzuerkennen und eine empathische Kommunikation mit Betroffenen zu gewährleisten, können den Zugang zu einer respektvollen und professionellen Versorgung verbessern.

Eine Sensibilisierung der breiten Öffentlichkeit für Post-COVID und die damit verbundenen Symptome ist wichtig. Zielgruppengerechte Informationskampagnen können helfen, die Vielseitigkeit und Ernsthaftigkeit der Symptome von Post-COVID zu verdeutlichen. Die Psychiatrie und Psychotherapie kann somit als zentraler Akteur wichtige Erfahrungen und Angebote in eine multiprofessionelle Zusammenarbeit bei postinfektiösen Folgeerkrankungen auch jenseits von Post-COVID einbringen und diese aktiv mitentwickeln.
